# QuilA-Adjuvanted *T. gondii* Lysate Antigens Trigger Robust Antibody and IFNγ^+^ T Cell Responses in Pigs Leading to Reduction in Parasite DNA in Tissues Upon Challenge Infection

**DOI:** 10.3389/fimmu.2019.02223

**Published:** 2019-09-20

**Authors:** Mizanur Rahman, Bert Devriendt, Ignacio Gisbert Algaba, Bavo Verhaegen, Pierre Dorny, Katelijne Dierick, Eric Cox

**Affiliations:** ^1^Laboratory of Immunology, Department of Virology, Parasitology and Immunology, Faculty of Veterinary Medicine, Ghent University, Merelbeke, Belgium; ^2^Sciensano, National Reference Center for Toxoplasmosis, Scientific Institute of Public Health, Communicable and Infectious Diseases, Brussels, Belgium; ^3^Department of Biomedical Sciences, Institute for Tropical Medicine, Antwerp, Belgium; ^4^Laboratory of Parasitology, Faculty of Veterinary Medicine, Ghent University, Merelbeke, Belgium

**Keywords:** *Toxoplasma gondii*, pigs, TLA vaccine, QuilA, magnetic capture-qPCR

## Abstract

*Toxoplasma gondii* is an intracellular parasite of all mammals and birds, responsible for toxoplasmosis. In healthy individuals *T. gondii* infections mostly remain asymptomatic, however this parasite causes severe morbidity and mortality in immunocompromised patients and congenital toxoplasmosis in pregnant women. The consumption of raw or undercooked pork is considered as an important risk factor to develop toxoplasmosis in humans. Since effective therapeutic interventions to treat toxoplasmosis are scarce, vaccination of meat producing animals may prevent *T. gondii* transmission to humans. Here, we evaluated the elicited immune responses and the efficacy of a potential vaccine candidate, generated by size fractionation of *T. gondii* lysate proteins, to reduce the parasite burden in tissues from experimentally *T. gondii* infected pigs as compared to vaccination with total lysate antigens (TLA). Our results show that both the vaccine candidate and the TLA immunization elicited strong serum IgG responses and elevated percentages of CD4^+^CD8^+^IFNγ^+^ T cells in *T. gondii* infected pigs. However, the TLA vaccine induced the strongest immune response and reduced the parasite DNA load below the detection limit in brain and skeletal muscle tissue in most animals. These findings might inform the development of novel vaccines to prevent *T. gondii* infections in livestock species and humans.

## Introduction

*Toxoplasma gondii* is a ubiquitous obligate intracellular apicomplexan parasite that may cause toxoplasmosis in endothermic animals including humans. The acute stage of infection is predominantly transient and transforms into a latent and chronic phase in which the parasite localizes within tissue cysts, mainly in the central nervous system (1). Although *T. gondii* infection is mostly asymptomatic in immunocompetent individuals, it might cause occasional illness, such as- muscle aches, tender lymph nodes, and eye problems (2). *T. gondii* develops tissue cysts in brain tissue, which has been associated with a vast array of psychiatric and behavioral disorders, such as schizophrenia and obsessive–compulsive disorder (3). Indeed, chronic *T. gondii* infections alter the morphology and function of neurons, dysregulating the release of neurotransmitters (3, 4). In addition, during pregnancy *T. gondii* infection can result in congenital toxoplasmosis, leading to abortion, stillbirth or birth defects (5).

Infection in humans generally occurs through the consumption of food or drinks contaminated with cat shed oocysts that sporulate in the environment, or through the consumption of raw or undercooked meat that contains tissue cysts (4–6). Other routes of infection are congenital transmission and organ transplantation (7). Raw and undercooked meat accounts for 30–63% of *T. gondii* infections in humans (8, 9) and according to the prevalence estimates of *T. gondii* in meat producing animals/meats, pork consumption was estimated to account for 12–15% of *T. gondii* infections in humans (10, 11). As such, preventing or treating *T. gondii* infections in pigs might help to reduce infection of humans.

Although toxoplasmosis is of great medical and veterinary importance, treatment of this disease is difficult. There are a number of drugs on the market, however their efficacy is variable when the treatment is started in an advanced stage of the infection and reactivation of the parasites may occur at any time (7, 12). As such, preventive interventions, like vaccination, that decrease the risk of contact with *T. gondii* are of utmost importance. Presently, only one commercial vaccine, Toxovax, that is based on the live attenuated *T. gondii* strain S48, has been licensed for sheep in some countries (13). However, this vaccine is expensive, causes adverse effects, has a short shelf-life and may revert to virulence (14). In addition, there is a knowledge gap whether this vaccine offers protection against *T. gondii* infection in other livestock species. Vaccination of pigs with *T. gondii* antigens incorporated in ISCOMs (immunostimulating complexes) elicited humoral immune responses, however it was not assessed if this vaccine affects the number of tissue cysts after challenge with *T. gondii* (15). Recently, it has been demonstrated that vaccination of pigs with *T. gondii* S48 strain might reduce the parasite load in skeletal muscles (16). For safety reasons and the issues described above, numerous research efforts have focused on the development of new vaccination strategies against toxoplasmosis, such as the development of DNA vaccines and subunit vaccines (17). These novel vaccine candidates need to elicit CD8^+^ cytotoxic T cell (CTL) responses, as the protection against *T. gondii* infection is mainly attributed to cellular immunity (14). However, there are two important obstacles hindering to meet this requirement. First, the immunogenicity of vaccine candidates should be enhanced. Purified *T. gondii* antigens exhibit a lower immunogenicity in contrast to live attenuated *T. gondii* vaccines as the latter are self-adjuvanting, mediated in part by cell wall and internal components. Therefore, adjuvants are often used in vaccine candidates to elicit better responses. For instance, in pigs a saponin-based adjuvant might be a good choice as it can promote systemic antibody responses, CTL responses and mucosal immune responses (18). A second hurdle comprises the limited information about potential vaccine antigens and their immunogenicity in pigs. To our knowledge, *T. gondii* elicits complex cellular immune responses that could differ considerably between host species and/or *T. gondii* strains. For example, during early *T. gondii* infection in sheep, the parasite triggers T cell independent IFNγ and IL-12 secretion, which are most likely crucial for resistance against the parasite. However, antigen-specific T cell dependent responses dominate around 8 days post infection (19). Apart from this, species-specific antibody responses were observed. In sheep infected with the *T. gondii* Prugniaud strain, antibodies appear against all tested *T. gondii* antigens (TLA, rGRA1, rGRA7, rEC2, or rMIC3), whereas in pigs only weak rGRA7-specific and even weaker TLA-specific antibody responses could be demonstrated when infected with the *T. gondii* IPB-Gangji strain (20, 21). *T. gondii* strains also differ considerably in their ability to trigger T cell responses. For instance, pigs inoculated with the *T. gondii* IPB-LR strain showed a significant increase in IFNγ^+^ T cell subsets (e.g., CD4^+^CD8α^−^, CD4^+^CD8α^dim^, and CD4^−^CD8α^bright^), while upon inoculation with the *T. gondii* IPB-Gangji strain the increase in the CD4^−^CD8α^bright^ T cells was clearly less pronounced (21). Nevertheless, a high dose of the IPB-Gangji strain elicited an immune response that resulted in a reduced parasite load, while a high dose of the IPB-LR strain did not affect the *T. gondii* distribution or load (21). In rats (intermediate host) and cats (definitive host) on the other hand a high dose of tachyzoites or bradyzoites resulted in a higher number of tissue cysts and a decreased survival rate of animals (22, 23). These discrepancies obviously complicate studies that aim to elucidate the potential of vaccine candidates to protect the host from *T. gondii* infections.

Here, we chose two different vaccine candidates in an effort to identify antigens which might provide protection against *T. gondii* infection in pigs upon their inclusion in vaccines. We opted for *T. gondii* lysate antigens (TLA) as vaccine candidate as TLA covers a wide range of potential protective antigens. To identify these potential protective antigens, proteins within TLA were size fractionated as this allows their unbiased separation. Upon evaluating the antigenicity of each size fraction, two selected fractions were combined as a second vaccine candidate. The humoral and cellular immune responses elicited by these two vaccine candidates as well as the parasite DNA load in selected tissues were evaluated.

## Materials and Methods

### *T. gondii* Strain

The *T. gondii* IPB-LR strain was used for experimental infections in pigs. This less pathogenic strain, originally isolated from pigs, belongs to genotype II, which is commonly present in the European pig population (21, 24, 25). There were two reasons to use this strain. Inoculation with the IPB-LR strain resulted in a more consistent infection and the parasite load in tissues could be reduced by a subsequent infection with the IPB-Gangji strain (21). The IPB-LR strain was maintained at the National Reference Laboratory for Toxoplasmosis (Sciensano, Brussels, Belgium) by passage in Swiss female mice. Tissue cysts were harvested from brain tissue, counted by phase-contrast microscopy and suspended in sterile phosphate buffered saline (PBS) at a concentration of 700 tissue cysts/ml (21). In addition, the *T. gondii* RH strain was used for the production of antigens.

### Vaccine Antigen Preparation

*T. gondii* lysate antigen (TLA) was prepared from tachyzoites of the RH-strain as previously described (17) and stored at −20°C until further use. To evaluate the protein content of the lysate, the bicinchoninic acid (BCA) reaction (Thermo Scientific Pierce BCA protein Assay Kit, Erembodegem, Belgium) was used. To separate TLA proteins according to their size a continuous-elution electrophoresis technique was used. TLA proteins (10 mg) were diluted (1:1) in loading buffer (0.04% w/v Bromophenol Blue, 0.04% w/v Xylene Cyanole FF, 6.66 % w/v Sucrose, 5% v/v ß mercaptoethanol) and denatured at 95°C for 5 min. Subsequently, the material was loaded on a 7.5% SDS-PAGE cylindrical gel (length 8 cm × diameter 3.7 cm) for continuous elution electrophoresis (PrepCell, Bio-Rad). During a run, TLA proteins migrated through the cylindrical gel matrix with running buffer (0.12 mM Tris-base, 0.96 M glycine, 0.5% SDS) as ring-shaped bands according to their molecular weight. The fractions were harvested in collection tubes (3.5 ml/tube) and aliquots (30 μl) were screened for the presence of TLA antigens by 7.5% SDS-PAGE and Western blotting. Upon blocking in PBS + 5% milk powder + 0.3% tween®80, transferred proteins were detected with *T. gondii*-specific pig serum (in house; 1/100 dilution) and goat anti-pig IgG-HRP (Bethyl, 1/1,000 dilution). Finally, western blot substrate (Pierce®ECL, Thermo Fisher Scientific) was applied as luminol enhancer and visualized using a ChemiDocMP Imager (BioRad, USA). According to the western blot results (Supplement Figure 1), we divided the TLA protein fractions in six pools according to their molecular weight: Pool 1 (20–40 kDa), Pool 2(40–55 kDa), Pool 3 (55–65 kDa), Pool 4 (65–80 kDa), Pool 5 (80–100 kDa), and Pool 6 (100–120 kDa). We further tested the immunogenicity of these pools in an antigen recall assay as described (21), which showed that pool 1 and 3 displayed the highest immunogenicity (Supplement Figure 2). Based on these results, we decided to combine pool 1 (20–40 kDa) and 3 (55–65 kDa) for vaccination (vaccine 1). At the same time, complete TLA was prepared as vaccine 2. In addition, both vaccines were concentrated and dialyzed to remove SDS with Amicon® filter units (cut-off = 10 kDa). Finally, both vaccine candidates were dissolved in PBS and sterilized by filtration (0.22 μm, low protein binding filters, Millex-GV). Protein concentrations were determined by BCA assay.

### Experimental Setup

**Figure 1:**
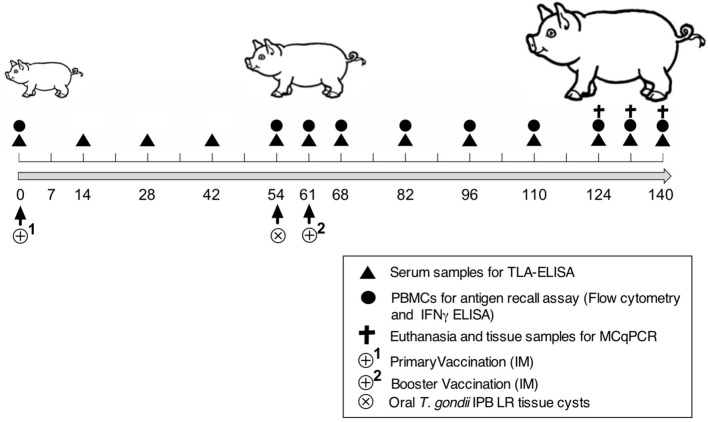
Scheme of the vaccination and challenge experiment. Experiment day indicated as numbers (0–140). TLA-ELISA: *T. gondii* lysate antigen-specific enzyme-linked immunosorbent assay. PBMCs, peripheral blood mononuclear cells. MCqPCR, magnetic capture real time quantitative polymerase chain reaction.

Nineteen 3-week-old piglets (Belgian Landrace × Large White) were confirmed to be *T. gondii* seronegative by the modified agglutination test (ToxoScreen DA, Biomérieux, Capronne, France) and an immunofluorescence test (Toxo-Spot IF, Biomérieux) as described (20). The piglets were randomly divided into four groups: control (*n* = 2), adjuvant (*n* = 2), vaccine 1 (*n* = 5), and vaccine 2 (*n* = 5). Figure 1 shows the timeline of the vaccination experiment. At day zero (D0) the piglets received an intramuscular (IM) immunization in the neck with vaccine 1 (500 μg antigens, 300 μg QuilA in 1.5 ml PBS), vaccine 2 (500 μg antigens, 300 μg QuilA in 1.5 ml PBS), adjuvant (300 μg QuilA) or were not immunized (control). At 54 days post immunization piglets were orally inoculated with 3,500 *T. gondii* tissue cysts and 61 days post primary immunization (7 days post inoculation) a booster vaccination was given. We followed this immunization scheme to obtain a booster response that could optimally interfere with the infection since our previous study using a heterologous challenge infection suggested that the early immune response was able to clear the parasite from tissues (21). Blood was sampled at the indicated time points in Figure 1 to evaluate serum antibody responses by ELISA and the function of peripheral immune cells in an *in vitro* recall assay. At the end of the study, pigs were euthanized by injecting sodium pentobarbital (20%, 0.125 ml/kg body weight, IV) and following exsanguination, tissue samples were collected to assess the parasite load.

### *T. gondii* Antigen-Specific ELISA

Blood samples were collected in vacuum tubes (Vacutest KIMA, Italy) from the vena jugularis according to the scheme shown in Figure 1. The blood samples were allowed to clot at room temperature for 30 min. Serum samples were collected upon centrifugation at 15,000 g for 10 min, aliquoted and stored at −20°C until further use. TLA, pool 1 and pool 3 antigen-specific serum IgG antibody responses were evaluated by ELISA as described (20). Briefly, 96-well microtiter plates were coated separately with TLA (5 μg/ml, Microbix Biosystems, Canada), pool 1 (5 μg/ml size fractionated antigens), and pool 3 (5 μg/ml size fractionated antigens), serial diluted serum samples were added and detected with HRP-conjugated anti-porcine IgG (Bethyl Laboratories Inc., Montgomery, Texas, USA) and ABTS [2,2′-azino-bis(3-ethylbenzothiazoline-6-sulphonic acid)] as a substrate (18). On each plate previously collected sera from positive and negative control animals, as established by IgM and IgG immunofluorescence assay (IFA), were included as a first line control. The absorbance at 405 nm was measured with a microplate reader (TECAN Spectra Fluor, Tecan Group Ltd., Männedorf, Switzerland) and the obtained data were analyzed in GraphPad Prism 6 software to discriminate IgG antibody responses between groups. Serum samples from infected animals were considered positive when exceeding the cut-off value (= mean OD_405_ negative controls + 3x the standard deviation). Antibody titers were calculated as the inverse of that dilution with a signal above the cut-off value.

### *In vitro* Recall Assay

Blood was sampled on heparin and PBMCs were isolated by Lymphoprep® gradient density centrifugation as described (21). PBMCs were seeded at 1 × 10^6^ cells/well in leukocyte medium (RPMI-1640, Thermo scientific, Merelbeke, Belgium) supplemented with fetal calf serum (10%, Greiner Bio-One, Merelbeke Belgium), non-essential amino acids (100 mM; Gibco), sodium pyruvate (100 μg/ml), L-glutamine (292 μg/ml; Gibco), penicillin (100 IU/ml; Gibco), streptomycin (100 μg/ml; Gibco), and kanamycin (100 μg/ml; Gibco) and were incubated with 20 μg/ml pool 1, pool 3, TLA or medium for 72 h. After incubation, 1 μl GolgiPlug (BD Biosciences) was added to each well for 6 h. Then, the cells were fixed and permeabilized using the Cytofix/Cytoperm kit (BD Biosciences) and stained using monoclonal antibodies (mAb) against CD3 (IgG1, clone PPT3), CD4 (IgG2b, clone 72–14-4), and CD8 (IgG2a, clone 11/295/33) and the secondary antibodies goat anti-mouse IgG1-PerCP-Cy5.5 (Santa Cruz Biotechnology, Dallas, Texas, USA), goat anti-mouse IgG2b-FITC (Southernbiotech, Birmingham, Alabama, USA) and goat anti-mouse IgG2a-Alexa Fluor 647 (ThermoFisher Scientific). Finally, for intracellular IFNγ staining, phycoerythrin (PE)-conjugated mAb against porcine IFNγ (mouse IgG1, BD Biosciences) was added. A minimum of 25,000 events were recorded within the CD3^+^ gate (Supplement Figure 4) and the percentage of IFNγ^+^ cells in the different lymphocyte subsets was determined using a CytoFLEX and Flow Cytometer software (both from Beckman Coulter). Finally, the percentage of IFNγ^+^ T cells were recalculated by subtracting the % IFNγ^+^ cells in unstimulated conditions from those with stimulation.

### IFNγ ELISA

IFNγ secretion was determined in the supernatant (1/10 dilution) of PBMCs cultured in medium or stimulated with antigens as described above with a sandwich ELISA using the swine-specific IFNγ antibody pair kit (ThermoFisher). The limit of detection of this ELISA was determined at 12.3 pg/ml.

### Magnetic Capture qPCR

The selection of tissue samples to assess parasite load in animals was based on our previous *T. gondii* IPB LR and/or Gangji strain infection study, in which we demonstrated that the tissues with the highest parasite load were detected in brain and heart followed by skeletal muscle intercostales, longissimus dorsi, psoas major, diaphragm, and gastrocnemius via Magnetic Capture qPCR (21).

The tissue samples from brain, heart and the skeletal muscles gastrocnemius and longissimus dorsi were collected from all pigs. The parasite load was determined by an ISO 17025 validated magnetic capture qPCR as previously described (26), in which the magnetic isolation of *T. gondii*-specific DNA from large tissue samples (>100 g) was combined with the sensitivity of qPCR. In addition, the developed MCqPCR has a sensitivity of 99% and a better limit of detection (26) than a previous MCqPCR (27).

All the samples with an exponential amplification curve crossing the threshold (Cq) were considered positive for *T. gondii*, while samples with no amplification curve for the *T. gondii* target, but amplification of the NCIAC (Not Competitive Internal Amplification Control) were considered negative. The detection limit of this method is 65.4 parasites per 100 g of tissue sample. For each round of samples, a positive control with a known number of parasites (calibrator) was included to correct for possible deviations due to manipulation errors. The number of parasites (n° p) was calculated according to the following formula:

(1) $$\log_{10}\left({\text{n}}^{{}^{\circ}}\text{p}\right)=\;\frac{{\text{Cq}}_{\text{value}}-44.75}{-3.0788}$$

The formula resulted from a standard curve established with known concentrations of parasites ranging from 100 to 10^5^ spiked in 100 g of muscle tissue samples or in 50 g of brain tissue (28). Log_10_(n° P) represents the log_10_-transformed parasitic load, while the Cq_value_ represents the point on the exponential amplification curve crossing the threshold.

### Data Analysis

The antibody responses, IFNγ response and *T. gondii* parasite load in tissue samples of the different groups are presented as mean ± SD. Individual group kinetics were analyzed with a repeated measures analysis of variance by the Friedman test and data between groups were analyzed by the Kruskal-Wallis test with a *post-hoc* analysis via Dunn's test. Multiple comparisons and computation of CIs along with significances were applied to discriminate between vaccinated and control groups. In all analyses *p* < 0.05 was considered statistically significant.

## Results

### *T. gondii* Vaccine Candidates Differ in Their Ability to Elicit Serum Antibody Responses

Previously, we obtained a strong indication that the early immune response following infection with the *T. gondii* IPB Gangji strain could clear the IPB LR strain in a heterologous challenge infection (21). Since we aimed to maximize the immune response of our experimental vaccines, we decided, based on this previous study, to orally challenge the vaccinated pigs with 3,500 tissue cysts of the *T. gondii* IPB LR strain seven days prior to the booster vaccination.

As potential vaccines we selected TLA (vaccine 2), this *T. gondii* lysate contains a lot of potential protective antigens. In an effort to enrich these potential protective antigens, the TLA proteins were size fractionated and their reactivity with *T. gondii* specific pig serum was confirmed (Supplement Figure 1). In addition, we observed that two pools, pool 1 and 3, elicited a robust T cell activation in an *in vitro* antigen recall assay (Supplement Figure 2). Based on these findings, pool 1 and pool 3 were selected to be included in vaccine 1. We then compared the ability of the two vaccine candidates to trigger immune responses in pigs.

**Figure 2:**
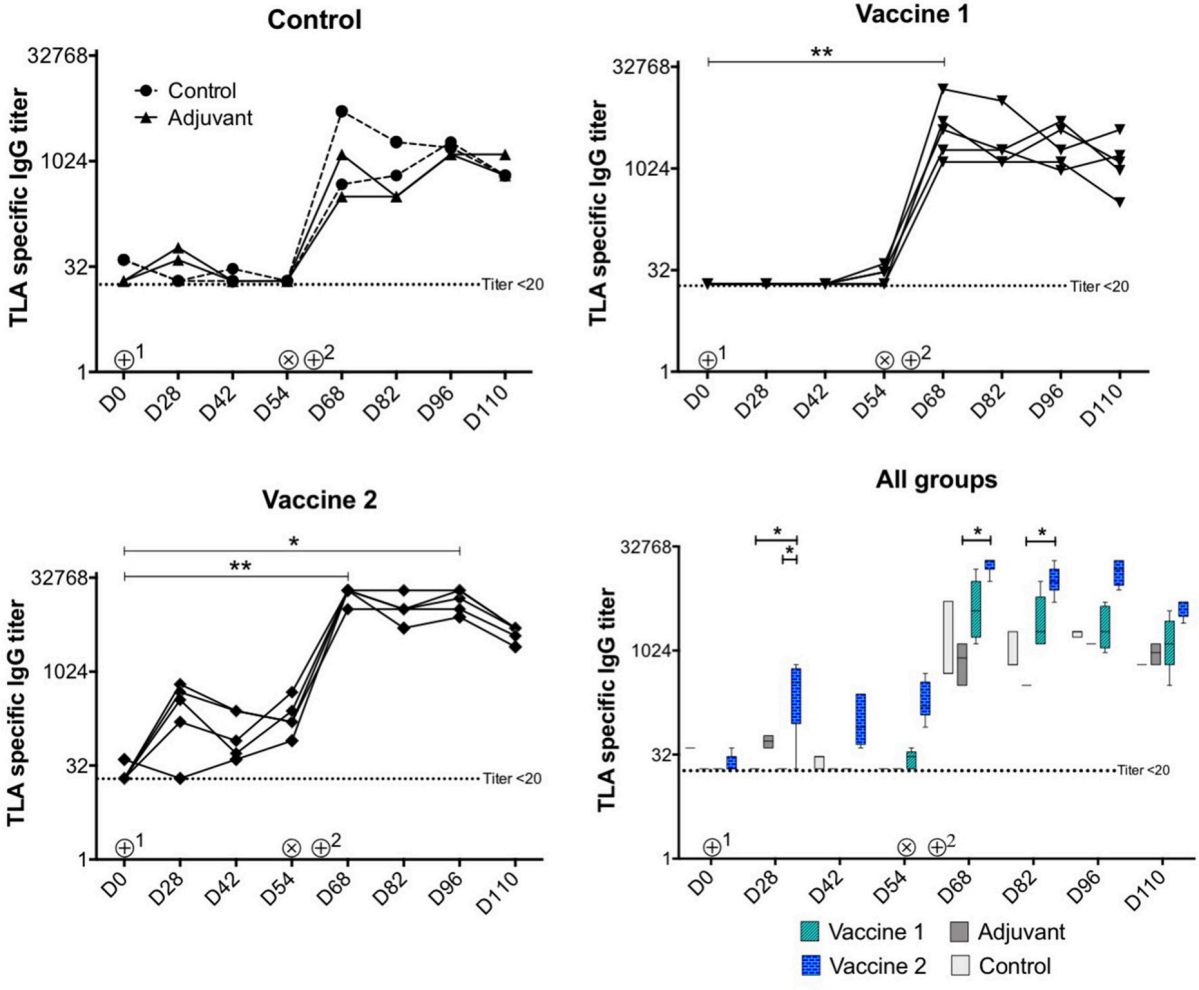
Kinetics of *T. gondii* specific serum IgG antibody titers upon vaccination and challenge infection. ⊕^1^ = primary vaccination, ⊕^2^ = booster vaccination, ⊗ = oral inoculation with *T. gondii* IPB LR strain tissue cysts. TLA, *T. gondii* lysate antigens; D, experiment day. The data are presented as the mean ± SD for each group. *P < 0.05; **P < 0.01. Symbols represent different animals.

As shown in Figure 2, upon intramuscular administration only vaccine 2 induced strong TLA-specific serum IgG responses (*P* = *0.02*) at D28 compared to vaccine 1 and control groups, respectively. In addition, TLA-specific IgG serum responses were significantly higher in vaccine 2 group at D68 (*P* = *0.03*) and at D82 (*P* = *0.02*) upon challenge infection compared to the adjuvant group. Upon challenge infection with *T. gondii* tissue cysts, TLA-specific serum IgG responses were significantly increased in vaccine 2 group at D68 (*P* = *0.008*) and at D96 (*P* = *0.019*) and in vaccine 1 group at D68 (*P* = *0.008*) compared to pre-challenge IgG levels and persisted throughout the study period, which corresponds with previous observations (29). Apart from this, the kinetics of the pool 1 and pool 3-specific serum IgG responses in all groups matched the TLA specific serum IgG response. Interestingly, in vaccine 1 group, the post-vaccination and pre-challenge serum IgG responses kinetics were clearly detectable, however this response was not detectable against TLA (Supplement Figure 3 and Figure 2).

### Vaccine Candidate 2 Induces a Different IFNγ Secretion Profile Upon Challenge Infection

**Figure 3:**
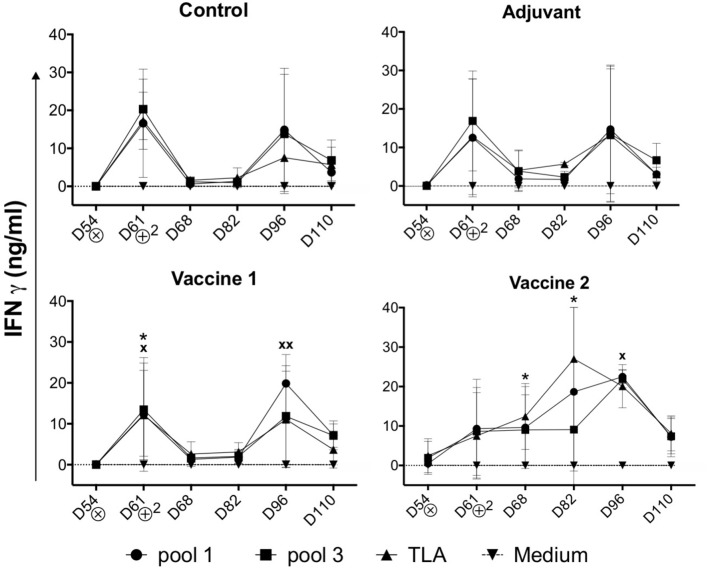
Kinetics of IFNγ secretion by PBMCs upon challenge infection. Pigs in all groups were inoculated with the *T. gondii* IPB LR strain at D54. ⊗ = Oral inoculation with *T. gondii* IPB LR strain tissue cysts. ⊕^2^ = Booster vaccination. The data are presented as the mean ±SD. * = TLA vs. medium, **x** = pool 1 vs. medium, * or x *P* < 0.05; xx *P* < 0.01.

In addition to the TLA-specific IgG responses, we also assessed the ability of the vaccine candidates to induce cellular immune responses. After challenge infection, PBMCs were isolated and stimulated *in vitro* with medium, TLA, pool 1 or pool 3 antigens and the IFNγ concentration in the supernatant was evaluated. In medium stimulated PBMCs the IFNγ level was below the limit of detection. In contrast, different antigens triggered IFNγ secretion by PBMCs in all groups (Figure 3). Interestingly, the IFNγ secretion profile in the vaccine 1 and two control groups (i.e., control and adjuvant groups) showed a bimodal response with a first peak of IFNγ secretion at D61 (7 days post infection) followed by a second peak at D96 (42 days post infection). We observed such a bimodal response in sheep following *T. gondii* infection, suggesting that the effect of vaccine 1 on the IFNγ response was minimal, which is supported by the similar response in the control and adjuvant only groups (19, 20). In the vaccine 1 group, TLA and pool 1 antigens triggered a significant IFNγ secretion (*P* = *0.037* and *P* = *0.044*, respectively) at D61, while at D96 this was only observed for pool 1 antigen (*P* = *0.006*). In contrast, the IFNγ secretion kinetics were completely different in the vaccine 2 group. The IFNγ secretion steadily increased from D54 until reaching a maximum at D82 in TLA-stimulated cells (*P* = *0.013*) and at D96 upon stimulation with pool 1 (*P* = *0.023*) antigens (Figure 3).

These findings accentuate that vaccine 2 induced IFNγ secretion by *T. gondii*-specific immune cells which maintained their robust IFNγ secretion during infection as compared to the control and vaccine 1 groups. In an effort to identify the source of this IFNγ, antigen-stimulated IFNγ producing PBMCs were phenotyped via FACS analysis.

### Different T Cell Subsets Produce IFNγ Upon Challenge Infection of the Vaccine Groups

**Figure 4:**
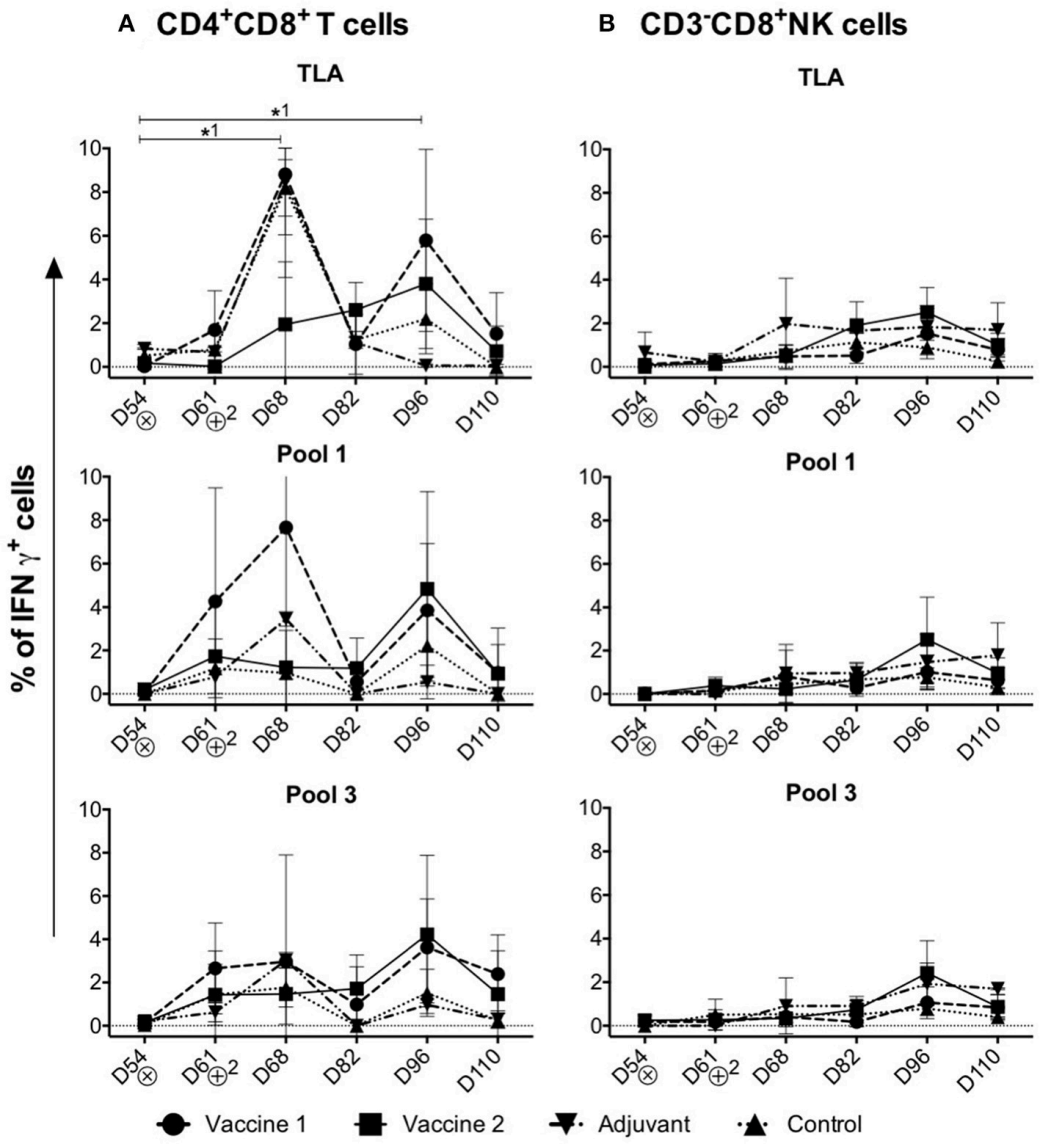
Kinetics of IFNγ^+^ T cell subsets in the control and vaccine groups upon challenge infection. **(A,B)** The percentage of CD4^+^CD8^+^IFNγ^+^ T cells and CD3^−^CD8^+^IFNγ^+^NK cells in TLA, Pool 1 and Pool 3 re-stimulated cells, respectively. The percentage of IFNγ^+^ T lymphocyte subsets is obtained by subtracting the %IFNγ^+^ cells following mock stimulation. ⊗ = Challenge infection with *T. gondii* IPB LR strain. The data are presented as the mean ± SD. *^1^ = vaccine 1. *^1^ *P* < 0.05.

Upon stimulation of PBMCs isolated from challenge infected piglets, the percentage of IFNγ^+^ cells in the different T cell subsets and NK cells was assessed (Figure 4). In addition to the conventional T cell subsets, pigs also have a large, unconventional CD4^+^CD8^+^ peripheral T cell population (30), which have been reported to differentiate from CD4^+^CD8^−^ cells upon antigen encounter (31). Indeed, this CD4^+^CD8^+^ T cell population exhibit properties of mature antigen-experienced memory/effector T cells, is characterized by the expression of IFNγ and plays a role in protection against viral infection (32, 33).

Throughout the study period, significant IFNγ^+^ T cell responses were detected in the CD4^+^CD8^+^ T cell subset (Figure 4A), but not in the CD4^−^CD8^+^ CTLs, CD4^+^CD8^−^ Th cells, CD4^−^CD8^−^ T cells or CD3^−^CD8^+^ IFNγ^+^ NK cells upon re-stimulation (Supplement Figures 5, 6A–C and Figure 4B).

Upon stimulation with TLA, the percentage of CD4^+^CD8^+^IFNγ^+^ T cells significantly increased in the vaccine 1 group at D68 (*P* = *0.02*) compared to D54. After a decline at D82, a second peak was observed in the vaccine 1 group at D96 which is also significant (*P* = *0.04*) compared to D54 (Figure 4A). On the other hand, in the vaccine 2 group, upon stimulation with TLA the percentage of CD4^+^CD8^+^IFNγ^+^ T cells slowly increased from D68 and reached its maximum at D96 (Figure 4A). A similar profile was observed upon stimulation with pool 1 and pool 3 antigens, although for the latter the first peak of IFNγ^+^ T cells at D68 was less prominent.

Collectively, these data show that the CD4^+^CD8^+^ T cells are the main IFNγ producers upon challenge infection of immunized pigs.

### Vaccine 2-Induced Immune Responses Prevented Dissemination of the Parasite to Brain Tissue in Infected Animals as Assessed by the Quantification of *T. gondii* DNA Load

**Figure 5:**
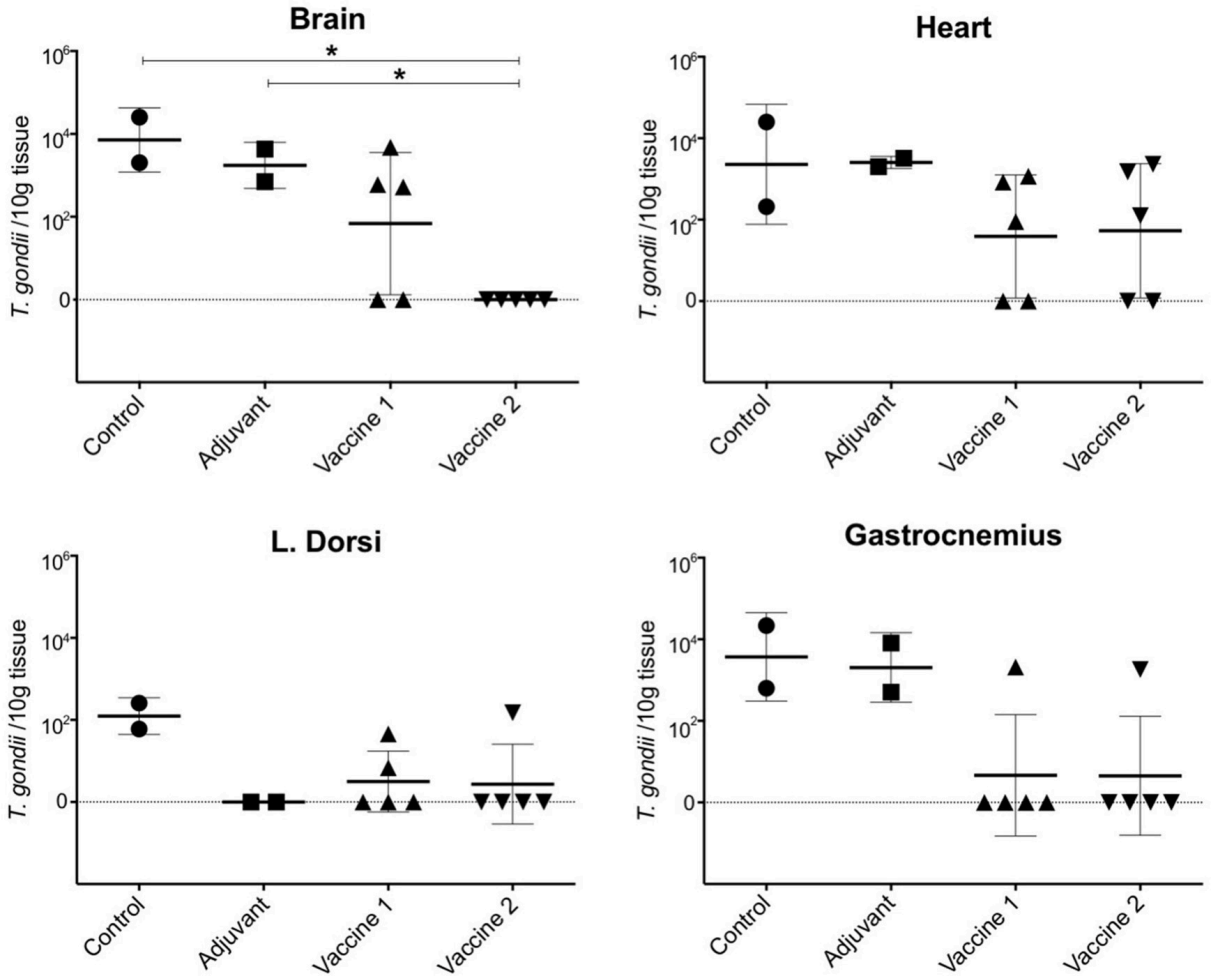
Presence of *T. gondii* parasite in the examined tissues upon challenge infection in the different vaccine groups. The data are presented as the number of *T. gondii* parasites/10 g tissue based on a formula resulting from a standard curve established with known concentrations of parasites ranging from 100 to 10^5^ spiked in 100 g of muscle or 50 g of brain tissue sample (28). The solid horizontal line indicates the mean ±SD. *P < 0.05.

In order to assess the effect of the vaccination on the ability of the animals to prevent dissemination of *T. gondii* parasites into the host tissues, representative tissue samples, such as brain, heart, m. longissimus dorsi, and m. gastrocnemius, were tested by magnetic capture qPCR (MC-qPCR) (26). According to the MCqPCR results, the control group displayed the highest parasite load in brain followed by heart, m. gastrocnemius and m. L. dorsi (Table 1), which corroborates our previous results (26). Remarkably, in the QuilA adjuvanted control group no parasite DNA was detected in the m. L. dorsi (Figure 5). In the vaccine 1 group, the parasite was not detected in brain tissue of 2/5 pigs, heart of 2/5 pigs, m. L. dorsi of 3/5 pigs, and m. gastrocnemius of 4/5 pigs. On the other hand, in vaccine 2 group, brain tissues of 5/5 pigs, heart of 2/5 pigs, m. L. dorsi of 4/5 pigs, and m. gastrocnemius of 4/5 pigs were parasite DNA negative.

**Table 1:** MCqPCR results obtained from different tissues of experimentally infected pigs (100 g muscle tissues or 50 g brain tissue).

**Group**		**Brain**	**Heart**	***L. dorsi***	**Gastrocnemius**
Control	Cp	32.42	29.05	37.13	29.25
	P-load	2022.26	25143.13	59.71	21650.05
	Cp	29.04	35.46	35.18	33.97
	P-load	25331.88	208.18	256.68	634.45
	**No. of positive pigs/total no. of pigs**	**2/2**	**2/2**	**2/2**	**2/2**
Adjuvant	Cp	33.83	31.79	-	34.27
	P-load	704.48	3239.38	0	506.94
	Cp	31.41	32.43	-	30.56
	P-load	4304.15	2007.19	0	8127.72
	**No. of positive pigs/total no. of pigs**	**2/2**	**2/2**	**0/2**	**2/2**
Vaccine 1	Cp	31.27	33.15	40.03	32.37
	P-load	4779.24	1171.47	6.82	2099.31
	Cp	34.22	33.60	37.49	-
	P-load	526.25	836.70	45.61	0
	Cp	-	36.59	-	-
	P-load	0	89.41	0	0
	Cp	40.07	-	-	-
	P-load	6.62	0	0	0
	Cp	-	-	-	-
	P-load	0	0	0	0
	**No. of positive pigs/total no. of pigs**	**3/5**	**3/5**	**2/5**	**1/5**
Vaccine 2	Cp	-	-	-	-
	P-load	0	0	0	0
	Cp	-	32.25	35.90	-
	P-load	0	2296.43	149.81	0
	Cp	-	32.83	-	32.56
	P-load	0	1488.22	0	1821.23
	Cp	-	-	-	-
	P-load	0	0	0	0
	Cp	-	36.14	-	-
	P-load	0	125.19	0	0
	**No. of positive pigs/total no. of pigs**	**0/5**	**3/5**	**1/5**	**1/5**

“-”, negative MCqPCR result; “o”, no parasite DNA detected; p-load, parasite load. Data are presented as the mean number of T. gondii parasite per 10 g of tissue.

## Discussion

The objective of this study was to evaluate the ability of *T. gondii* lysate fractions as vaccine candidates to elicit appropriate immune responses, which could protect pigs from *T. gondii* infection. To design a vaccine candidate, two issues were considered: the choice of antigen and adjuvant. As protection against *T. gondii* infection requires a potent cellular immune response, we adjuvanted the vaccine candidates with QuilA. The latter elicits robust antibody and cellular immune responses to a broad range of viral, bacterial, parasitic and tumor antigens (34, 35). Second, we wanted to include *T. gondii* antigens in the vaccine candidate that were able to trigger antibody and cellular immune responses. For this, *T. gondii* lysate proteins were size fractionated and pooled according to their molecular weight. Based on their immunogenicity and their ability to elicit IFNγ secretion in an *in vitro* antigen recall assay, pool 1 and 3 were combined and used as vaccine candidate 1. For comparison, complete TLA was used as vaccine 2.

Despite our selection procedure, only vaccine 2 elicited TLA-specific serum IgG responses upon intramuscular administration of a single dose (Figure 2), whereas both vaccines induced a pool 1- and a lower pool 3-specific serum IgG response (Supplement Figure 3). This might be explained by the fact that pool 1 and 3 antigens in vaccine 1 are denatured during processing. However, that both pools form only a fraction of antigens in TLA might also have contributed to the absence of TLA-specific IgG response after a single dose of vaccine 1. Nevertheless, vaccine 1 probably still primed the immune system as the antibody responses upon challenge infection and booster vaccination were higher as compared to those of the control groups. Likewise, in vaccine 2 group the post-challenge IgG titers were significantly higher compared to the control group. These high *T. gondii* specific IgG titers upon challenge infection in all groups, which persisted till the end of the study, are in line with previous findings, which demonstrated that irrespective of the *T. gondii* strain and age of the pigs, parasite-specific IgG appeared day 7–14 post infection and persisted throughout life (29).

Although the role of antibodies in protection against *T. gondii* is largely unknown, cellular immune responses, and more specific IFNγ production, are strongly correlated with resistance against *T. gondii* infection (36). In our previous study, we showed IFNγ production by CD4^+^ Th cells, CD8^+^ CTLs and CD4^+^CD8^+^ memory T cells of *T. gondii*-infected piglets in an *in vitro* antigen recall assay (21). Here, we showed that upon infection of the control and vaccine 1 group, CD4^+^CD8^+^ T cells secreted IFNγ upon TLA stimulation in a bimodal response, while in the vaccine 2 group this IFNγ production by CD4^+^CD8^+^ T cells gradually increased until D96 of the study. These results indicate that vaccine 2 might have induced *T. gondii*-specific IFNγ^+^ T cells which upon challenge and booster vaccination of the pigs differentiated into effector memory T cells (37). In contrast, the bimodal IFNγ response in the control and vaccine 1 group might be explained by an interplay between the life cycle of the parasite and the induced immune responses. Upon oral ingestion, tissue cysts release bradyzoites in the intestine, which transform to tachyzoites. The latter invade and replicate the surrounding tissues, from which they eventually disseminate throughout the host to other organs. IFNγ drives the reversion of these tachyzoites into bradyzoites, which then form cysts to hide from the immune system (38, 39). It seems that in the control and vaccine 1 group, infection triggered IFNγ-producing T cells, which resulted in tissue cysts. The subsequent absence of *T. gondii* antigens might have prevented their development into long-lived memory T cells and upon disappearance of the short-lived *T. gondii*-specific IFNγ-producing T cells, tissue cysts released their content, resulting in a second wave of tachyzoites and the associated IFNγ response. Alternatively, the decline in IFNγ-producing T cells in blood upon infection of control and vaccine 1 group might hint at their migration to infected tissues. The different kinetics of IFNγ production in the vaccine 2 group might be explained by the broader antigenic range of vaccine 2, resulting in *T. gondii*-specific antibodies and T cells which might have controlled the parasite at the initial site of infection.

This difference in IFNγ response might explain the different ability of the two vaccines to prevent the dissemination of the parasite DNA from the intestinal tissues to the other organs upon challenge infection.

Here, the parasite load in challenge infected animals was assessed by MC-qPCR. Although this technique does not provide information on the viability of the parasites, it does allow to quantify *T. gondii* DNA and to assess vaccine efficacy. In contrast to the control groups and vaccine 1 group, parasite DNA was not detected in brain tissue and skeletal muscle tissue (4/5 animals were negative) in vaccine 2 group, implying that this vaccine candidate might have the ability to control *T. gondii* dissemination to these tissues. Hence, it might be interesting to investigate if the induced immune response after the first immunization suffices for this. In both vaccine groups, most animals were positive for parasite DNA in heart tissue. Heart muscles are well-connected to the oxygenated blood circulation which ultimately could help *T. gondii* growth and survival (40). Alternatively, the induced immune responses might have been inadequate to prevent infection of cardiac cells with *T. gondii* for reasons yet unknown. Surprisingly, in the QuilA adjuvanted control group no parasite was detected in the m. L. dorsi. These results might indicate that the activation of the immune system by QuilA accelerated *T. gondii*-specific immune responses upon challenge infection, which were able to prevent the dissemination of the parasite in this tissue. These findings warrant further research.

In conclusion, infection of pigs with *T. gondii* is an interesting model to evaluate vaccine strategies. Here, vaccine 2 seems to be a better vaccine candidate as compared to vaccine 1 to elicit *T. gondii* specific antibody and T cell responses which prevent dissemination of the parasite to the brain upon oral infection in pigs. However, further research is needed to confirm that the reduction in parasite DNA load is correlated with decreased parasite infectivity of tissues and to optimize the efficacy, the dose as well as the route of administration.

## Data Availability

All datasets generated for this study are included in the manuscript/Supplementary Files.

## Ethics Statement

The animal procedures were approved in accordance with the recommendations of the ethical standards defined by the EU legislation on the use of laboratory animals (2010/63/EU) and in accordance with the Belgian law (Royal Decree 29/5/2013 and 30/11/2001). In addition, the experiment protocols were approved by the Ethical Committee of the Faculty of Veterinary Medicine and the Faculty of Bioscience Engineering, Ghent University (EC 2009/149) and by the Ethical Committee of Sciensano (176 20140704-01).

## Author Contributions

MR and EC designed the study. MR performed the experiments, acquired and analyzed the data, and drafted the manuscript. BD analyzed the data and wrote the manuscript. IG and BV helped to perform MCqPCR and revised the manuscript. PD and KD helped to design the study, gave valuable input, and revised the manuscript. EC analyzed the data and reviewed the manuscript.

### Conflict of Interest Statement

The authors declare that the research was conducted in the absence of any commercial or financial relationships that could be construed as a potential conflict of interest.

## Abbreviations

Cp: Crossing point (also known as Ct or Cq)
ELISA: Enzyme-Linked ImmunoSorbent Assay
IFA: Indirect Immunofluorescence Assay
MAT: Modified Agglutination Test
MC-qPCR: Magnetic Capture- Quantitative Polymerase Chain Reaction
PBS: Phosphate-buffered saline
ng/ml: Nanogram/milliliter
TLA: Total Lysate Antigen
FACS: Fluorescence-activated cell sorting
IFN-γ: Interferon gamma
PBMCs: Peripheral blood mononuclear cells
LOD: Limit of detection
SD: Standard deviation
CD: Cluster of differentiation
NK cells: Natural killer cells
IL: Interleukin
APCs: Antigen presenting cells
CTLs: Cytotoxic T lymphocytes
Th cells: T helper cells
CCE: Continuous elution electrophoresis
kDa: Kilodalton
MW: Molecular Weight.
