# Supplementary figures and images for: QuilA-Adjuvanted T. gondii Lysate Antigens Trigger Robust Antibody and IFNγ+ T Cell Responses in Pigs Leading to Reduction in Parasite DNA in Tissues Upon Challenge Infection
Source: Front Immunol. 2019 Sep 20;10:2223. doi: 10.3389/fimmu.2019.02223 (PMC6763570; doi:10.3389/fimmu.2019.02223)

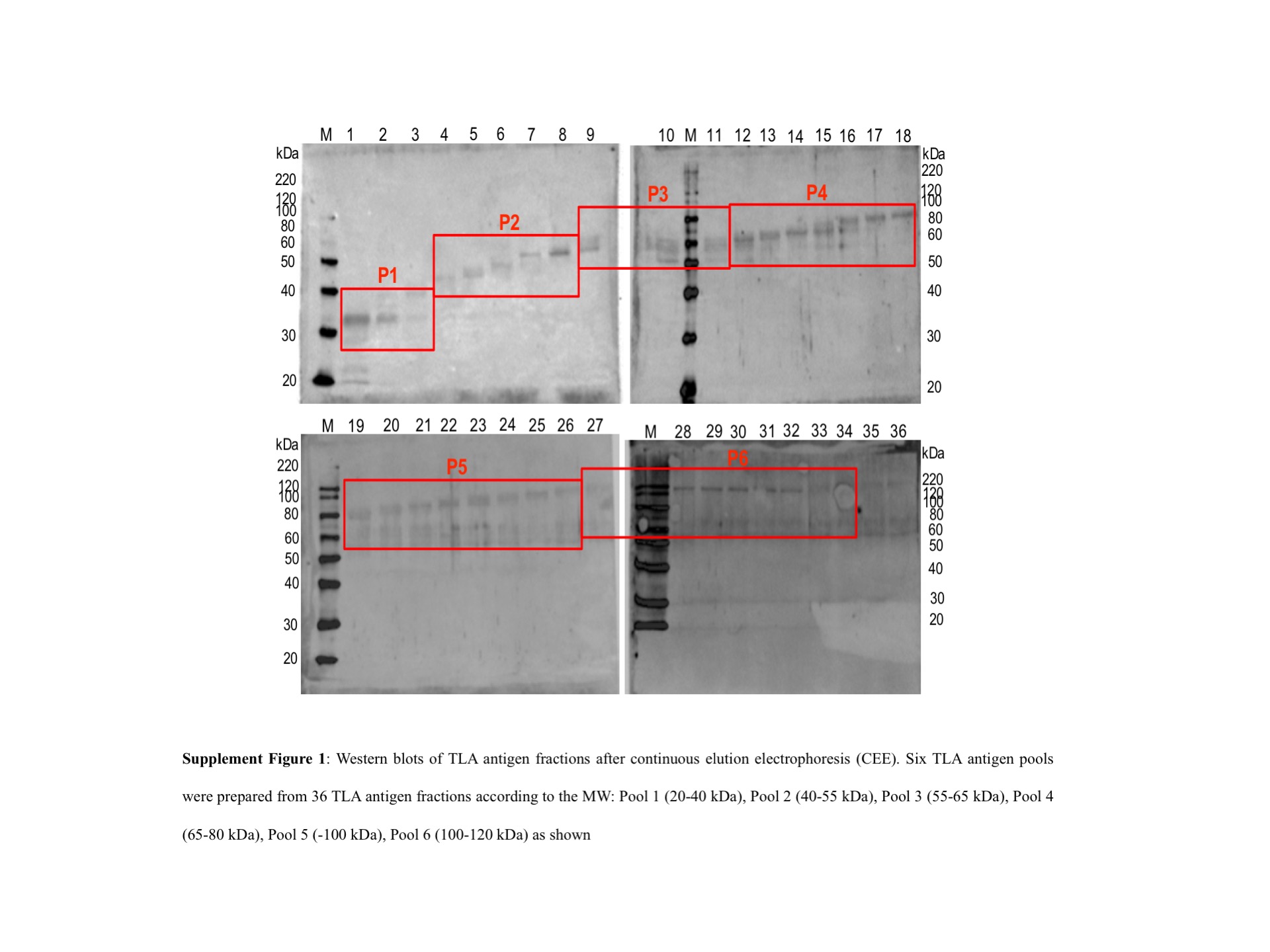

Supplement: Supplementary file 1 [file Image_1.JPEG]

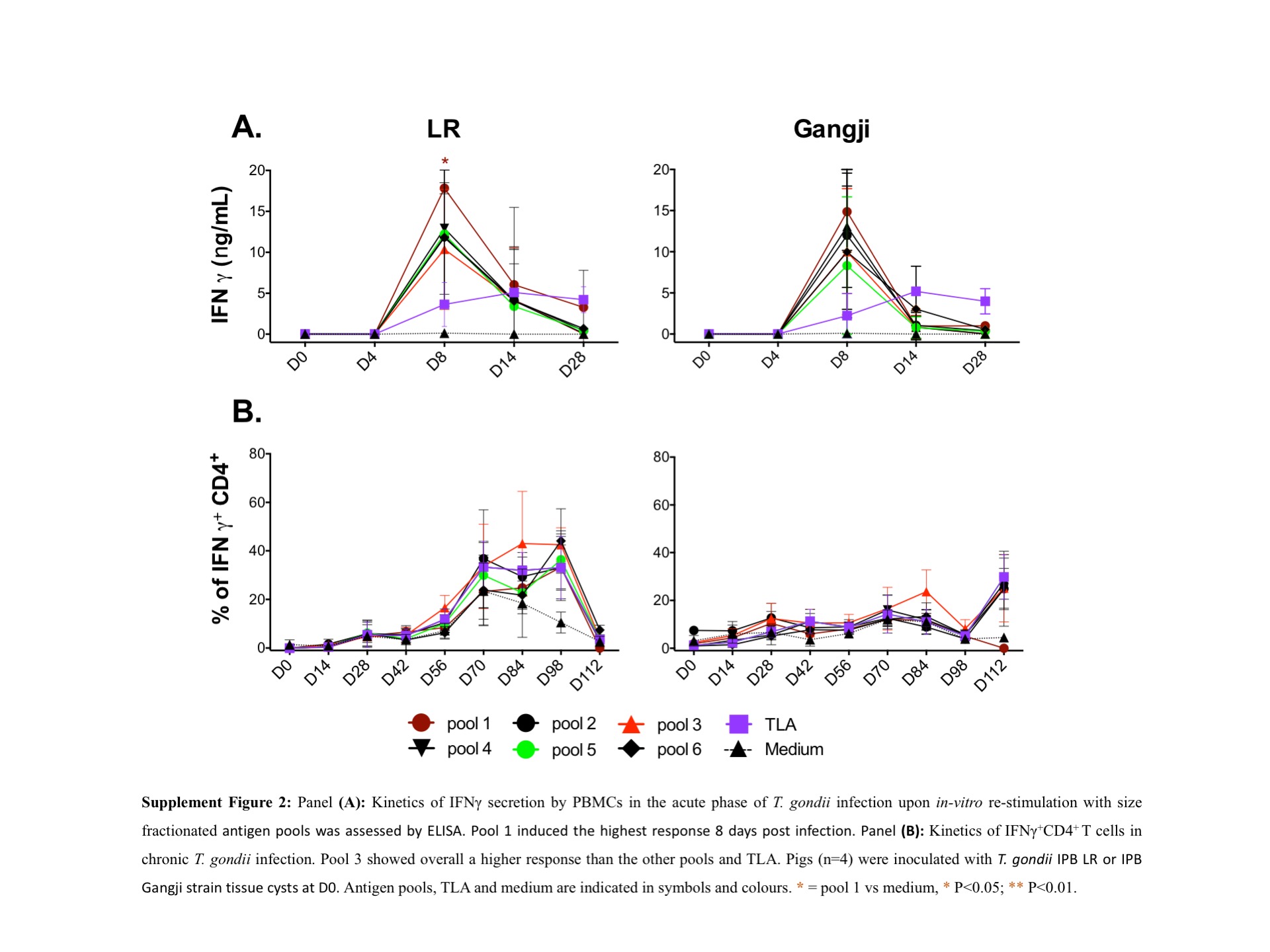

Supplement: Supplementary file 2 [file Image_2.JPEG]

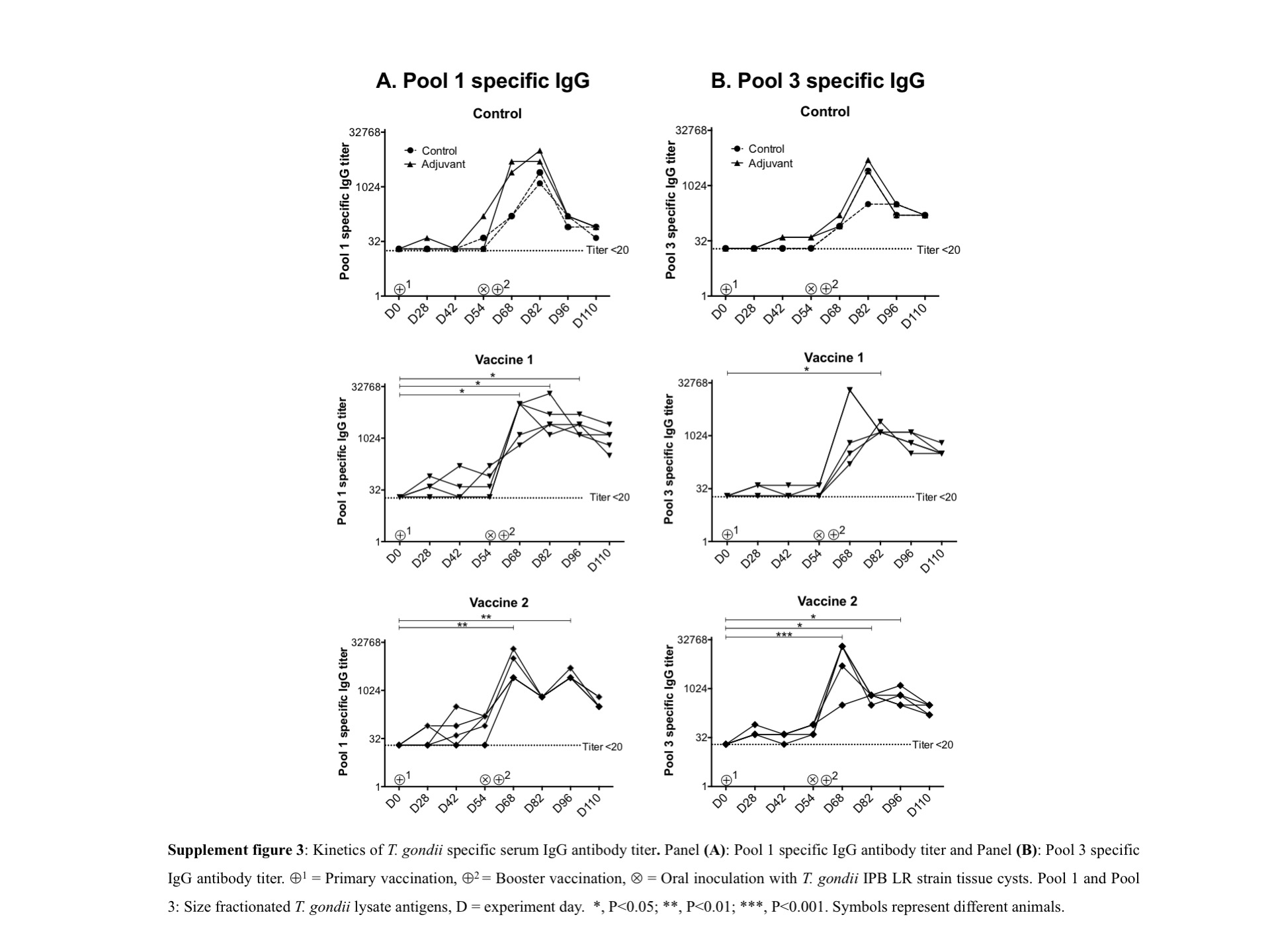

Supplement: Supplementary file 3 [file Image_3.JPEG]

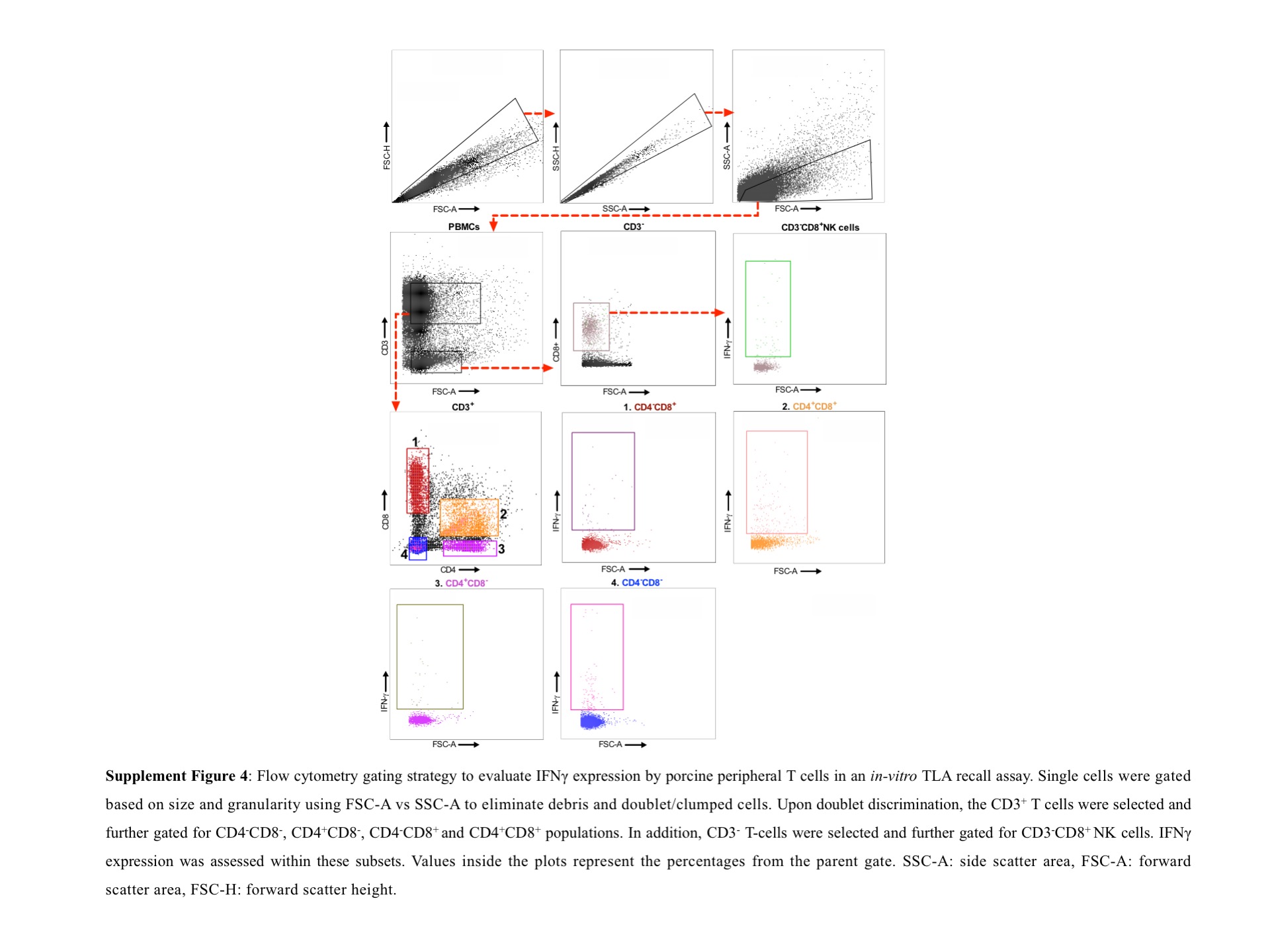

Supplement: Supplementary file 4 [file Image_4.JPEG]

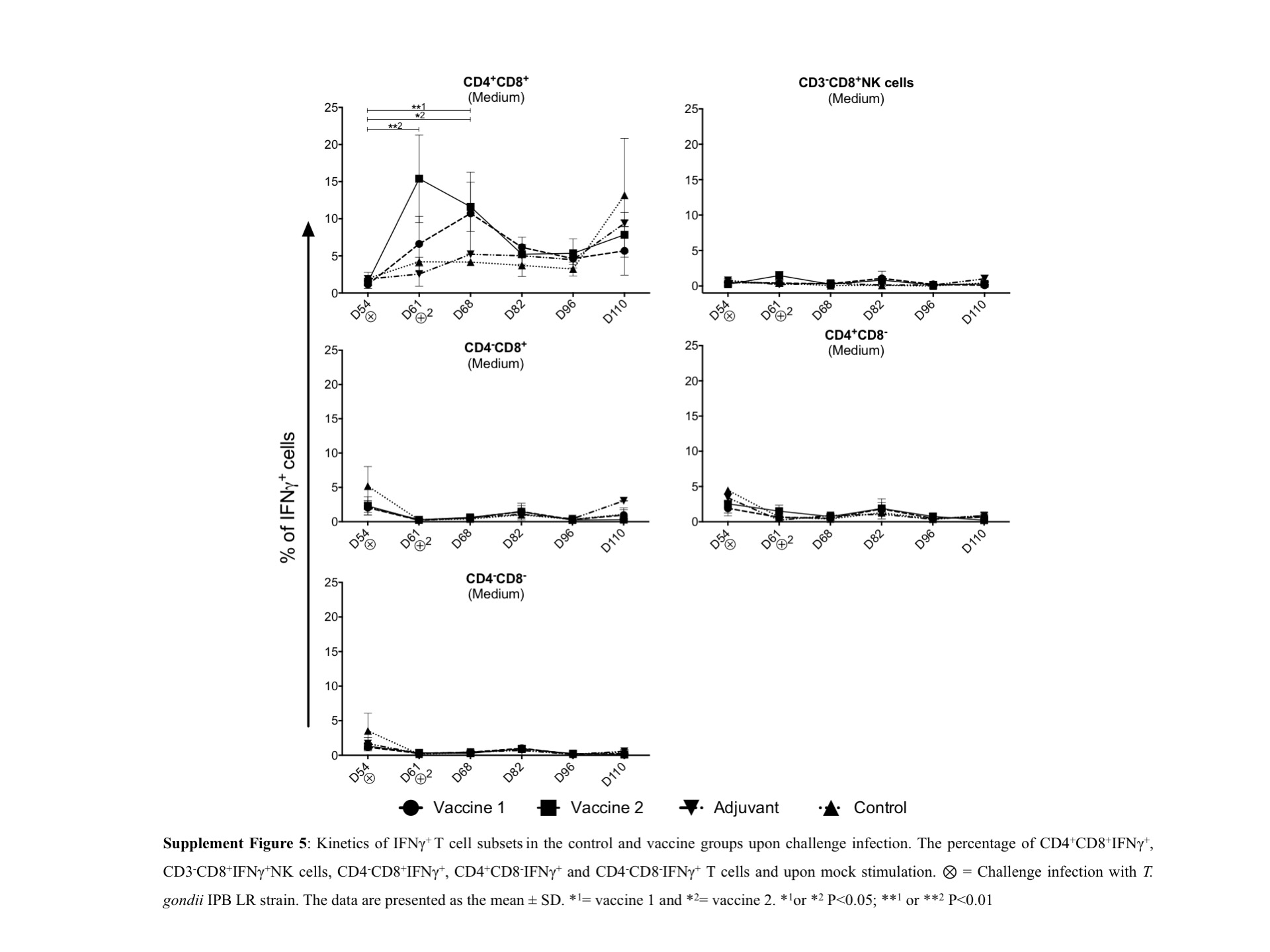

Supplement: Supplementary file 5 [file Image_5.JPEG]

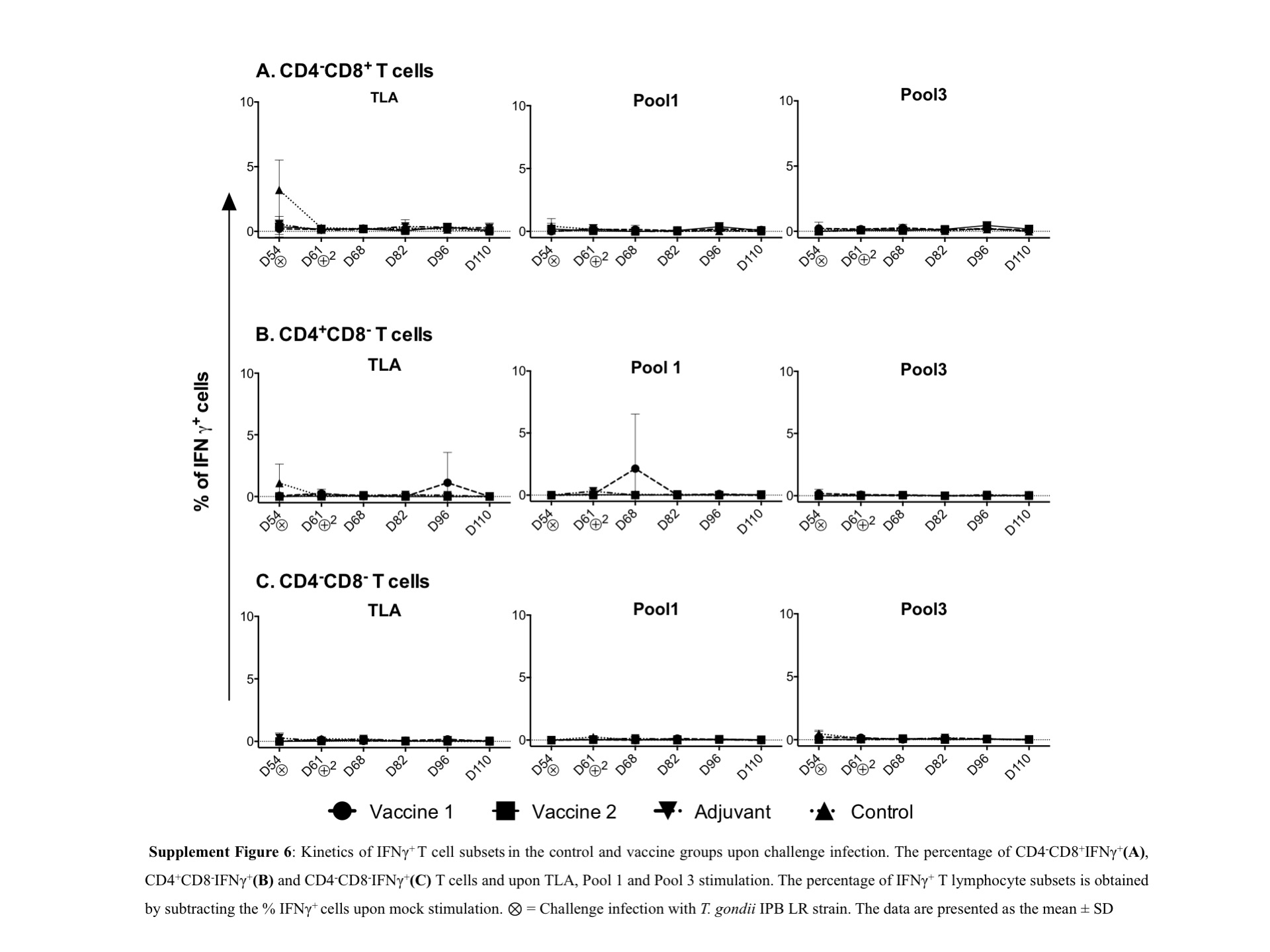

Supplement: Supplementary file 6 [file Image_6.JPEG]
